# Solution Structures of PPAR**γ**2/RXR****α**** Complexes

**DOI:** 10.1155/2012/701412

**Published:** 2012-12-18

**Authors:** Judit Osz, Maxim V. Pethoukhov, Serena Sirigu, Dmitri I. Svergun, Dino Moras, Natacha Rochel

**Affiliations:** ^1^Department of Integrative Structural Biology, Institut de Génétique et de Biologie Moléculaire et Cellulaire (IGBMC), Centre National de Recherche Scientifique (CNRS) UMR 7104, Institut National de Santé et de Recherche Médicale (INSERM) U964, Université de Strasbourg, 67404 Illkirch, France; ^2^The European Molecular Biology Laboratory, Hamburg Outstation, 22603 Hamburg, Germany

## Abstract

PPAR**γ** is a key regulator of glucose homeostasis and insulin sensitization. PPAR**γ** must heterodimerize with its dimeric partner, the retinoid X receptor (RXR), to bind DNA and associated coactivators such as p160 family members or PGC-1**α** to regulate gene networks. To understand how coactivators are recognized by the functional heterodimer PPAR**γ**/RXR**α** and to determine the topological organization of the complexes, we performed a structural study using small angle X-ray scattering of PPAR**γ**/RXR**α** in complex with DNA from regulated gene and the TIF2 receptor interacting domain (RID). The solution structures reveal an asymmetry of the overall structure due to the crucial role of the DNA in positioning the heterodimer and indicate asymmetrical binding of TIF2 to the heterodimer.

## 1. Introduction

PPAR*γ*, a member of the nuclear receptor family, is a key regulator of adipocyte differentiation and is involved in glucose homeostasis and insulin sensitization (reviewed in [1, 2]). PPAR*γ* together with the CCAAT/enhancer-binding proteins had been identified as key transcription factors of driving fat cell differentiation (reviewed in [3]). PPAR*γ* is absolutely required for both white and brown fat cell development. Several PPAR*γ* coregulators have also been shown to affect positively or negatively this differentiation (reviewed in [4]).

PPAR*γ* is activated through the binding of diverse ligands including natural fatty acid derivatives and nonsteroidal drugs and is the target of therapeutically active antidiabetics such as rosiglitazone (reviewed in [5]). Furthermore, cdk-5 phosphorylation of PPAR*γ* leads to deregulation of some genes involved in metabolism [6]. 

**Figure 1:**
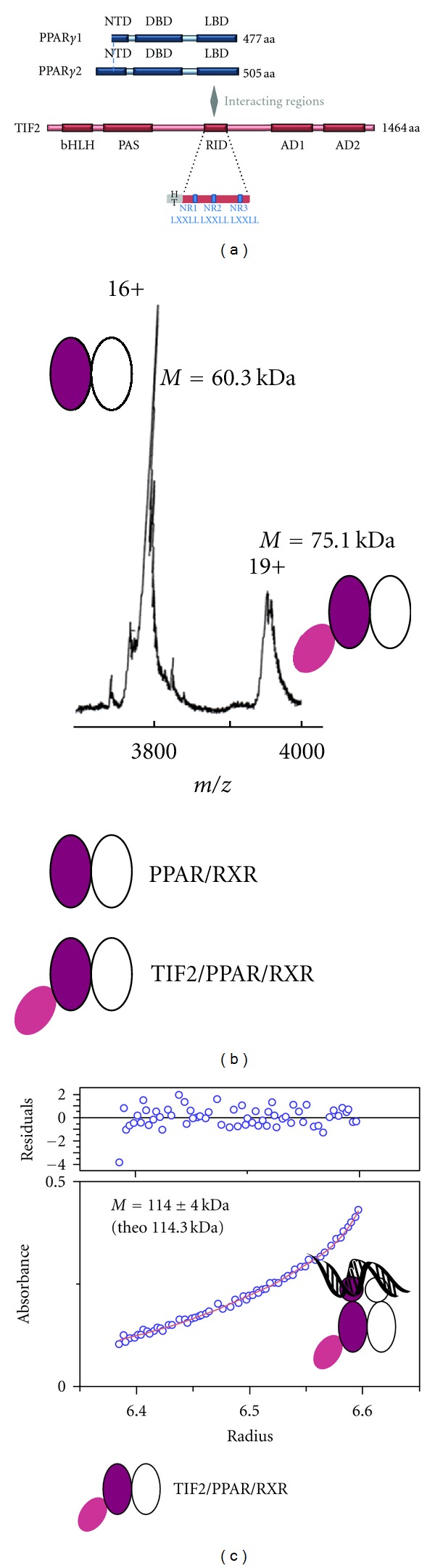
Biophysical characterization of the stoichiometry of the TIF2 RID/PPAR*γ*/RXR complexes. (a) Structural organization of hPPAR*γ*1, hPPAR*γ*2, and hTIF2. (b) ESI mass spectra of TIF2 RID/PPAR*γ*/RXR LBDs recorded under nondenaturing conditions in 200 mM ammonium acetate at pH = 7.4. The different charge states of the proteins are indicated above the peaks. The calculated molecular mass of the first peak corresponds to PPAR*γ*/RXR*α* LBDs and the second one to the complex containing one PPAR*γ*/RXR*α* LBDs dimer and one TIF2 RID. (c) Sedimentation equilibrium experiments. Best fits of experimental data for TIF2 RID/PPAR*γ*ΔNTD/RXRΔNTD at 12,000 rpm with the self-association methods (SedPhat program). Sedimentation equilibrium data agrees with one TIF2 RID bound to PPAR*γ*ΔNTD/RXRΔNTD.

The actions of PPAR*γ* are mediated by 2 isoforms that result from alternative splicing. PPAR*γ*2 is 28 amino acids longer at the N-terminal end (Figure 1(a)) and is mainly expressed in adipocyte cells, while PPAR*γ*1 is ubiquitously expressed. Interestingly, PPAR*γ*2 is ten times more active in ligand-independent transcriptional activation than PPAR*γ*1 [7, 8].

PPAR*γ* as the other nuclear receptors is a modular protein with a DNA binding domain (DBD) and a ligand binding domain (LBD). It is active as a heterodimer with the retinoid X nuclear receptor (RXR). The PPAR/RXR heterodimer recognizes specific DNA sequences called PPAR response elements (PPRE), composed of imperfect direct repeats separated by one nucleotide (DR1) and an extended 5′ half-site hexanucleotide [8]. Both the nonsymmetrical DR1 and the 5′ flanking sequence contribute to the selective binding of PPAR. PPAR binds the 5′ hexanucleotide and the 5′ flanking region, while RXR binds the 3′ half-site. To be fully active, PPARg2/RXR must be associated with coactivators which include p300/CBP, p160 members, PGC-1*α*, as well as Med1 (reviewed in [9, 10]). These coactivators are themselves highly regulated at the transcriptional and posttranscriptional levels. 

Structurally, most of our current knowledge on the molecular basis of the mechanism of action of NRs is based on the X-ray and NMR structures of isolated DNA and ligand binding domains. The crystal structures of various NR LBDs with coactivator peptides have revealed a conserved binding mode of the coactivators to NRs. Agonist ligands trigger a conformational change of the NR LBDs allowing a leucine-rich interacting motif (LXXLL motif) present in the coactivator sequence that forms an *α*-helix to interact through a charge clamp and hydrophobic interactions at the LBD surface [11–13]. Coactivators such as p160 or PGC-1*α* contain several LXXLL motifs (NR boxes) involved in specific binding to NRs and other transcription factors. For TIF2 (NCoA2, p160 member), 3 NR boxes are found in a central domain that interacts with NRs and is called the receptor interacting domain (RID) (Figure 1(a)). The third motif has been shown to preferentially bind to PPAR*γ* [14].

Extensive studies on structure-function relationships have been done on PPAR*γ* LBD monomers or dimers and have provided information on conformational change induced by various ligands. The structures of full-length PPAR*γ*/RXR complexes have also been studied by X-ray crystallography [15] and by small-angle X-ray scattering (SAXS) [16, 17], although the N-terminal domain (NTD) was not visible in the electron density map. Remaining questions concern the mode of recognition of coactivator domains by PPAR*γ*/RXR*α* complexes. To understand how TIF2 RID is recognized by PPAR*γ*/RXR*α* and the topological organization of the complex, we performed a structural analysis in solution by SAXS of PPAR*γ*/RXR*α* LBD in complexes with TIF2 RID and of PPAR*γ*2/RXR*α* full-length or depleted of their N-terminal domain (ΔNTD) bound to PPRE and TIF2 RID protein.

## 2. Materials and Methods

### 2.1. Cloning, Protein Expression, and Purification

The HsPPAR*γ* LBD (203–477), HsPPAR*γ*ΔNTD (135–505) and HsTIF-2 (632–772) were expressed as hexahistidine fusion proteins. HsRXR*α* LBD (223–462) and HsRXR*α*ΔNTD (130–462), were cloned into pACYC plasmid encoding nontagged proteins. The nuclear receptor heterodimers were coexpressed in *E. coli* BL21 (DE3) cells. Full-length HsPPAR*γ*2 (1–505) and HsRXR*α* (1–462) were coexpressed in Sf9 insect cells as N-ter hexahistidine and Flag tagged fusion proteins, respectively. The PPAR/RXR dimers and the coactivator TIF-2 RID were purified by affinity chromatography and gel filtration as described [16, 18]. Ligands (*CD3254* for RXR and rosiglitazone for PPAR) were added in a 2-fold excess to saturate the receptors. The DNA (GAAACTAGGGTAAAGGTCAG/CTTTGATCCCATTTCCAGTC) was added in a 1.2-fold excess to the dimers, and the complex was gel-filtrated on a Superdex S200 (16/60 or 10/300, GE Healthcare). For the PPAR/RXR/TIF2 RID complexes, TIF2 was added in a 2-fold excess and gel-filtrated on Superdex S200 (16/60 or 10/300, GE Healthcare). Protein samples were concentrated using Amicon Ultra centrifugal filter units (Millipore). Purity and homogeneity of the protein were assessed by SDS-PAGE, and complex formation was monitored by native PAGE. The final buffer for PPAR/RXR LBDs was Tris 20 mM pH 7.5, NaCl 200 mM, and DTT 5 mM and for PPAR*γ*/RXR*α*/DNA complexes Tris 20 mM pH 7.5, NaCl 75 mM, KCl 75 mM, MgSO_4_ 4 mM, Glycerol 5%, and Chaps 2 mM. 

### 2.2. Ultracentrifugation Equilibrium Sedimentation

For sedimentation equilibrium experiments, samples were spun at 12,000 rpm and systems were first allowed to equilibrate for 12 hours before absorbance profiles were compared at different times to ensure that system had reached equilibrium. Using nonlinear least-squares analysis, these datasets were fitted using single component model and several equilibrium models with Sedphat program.

### 2.3. Electrospray Ionization Mass Spectrometry. 

Prior to ESI-MS analysis, samples were desalted on Zeba Spin desalting columns (Pierce) in 150 mM ammonium acetate (pH 8.0). ESI-MS measurements were performed on an electrospray time-of-flight mass spectrometer (MicrOTOF, Bruker Daltonic, Germany). Purity and homogeneity of the proteins were verified by mass spectrometry in denaturing conditions (samples were diluted at 2 pmol/*μ*L in a 1 : 1 water-acetonitrile mixture (v/v) acidified with 1% formic acid). The mass measurements of the noncovalent complexes were performed in ammonium acetate (200 mM; pH 8.0). Samples were diluted to 8 pmol/mL in the previous buffer and continuously infused into the ESI ion source at a flow rate of 3 mL/min through a Harvard syringe pump (Harvard Apparatus model 11). A careful optimization of the interface parameters was performed to obtain the best sensitivity and spectrum quality without affecting the noncovalent complexes stability. In particular, the capillary exit (CE) ranged from 60 to 150 V with a vacuum interface pressure of 2.3 mbar and was set to 80 V. 

### 2.4. SAXS Experiments and Data Processing. 

Synchrotron X-ray solution scattering data were collected at the X33 beamline (DESY, Hamburg) [19] using a PILATUS detector at a sample-detector distance of 2.7 m, covering the range of momentum transfer 0.01 < *q* < 0.6 Å^−1^ (*q* = 4*π*sin(*θ*)/*λ*, where 2*θ* is the scattering angle and *λ* = 0.15 nm is the X-ray wavelength) in eight frames (15 seconds each) to check for possible radiation damage. All scattering measurements were carried out at 10°C with automated filling, and samples were measured at several concentrations. 

SAXS experiments were also conducted on the SWING beamline at SOLEIL Synchrotron (Gif-sur-Yvette, France), using a 17 × 17 cm^2^ low-noise Aviex CCD detector positioned at a distance of 2.107 m from the sample. Sample solutions were circulated in a thermostated Quartz capillary with a diameter of 1.5 mm and 10 *μ*m wall thickness, positioned within a vacuum chamber. Fifty frames of 2 s each were collected, normalized to the transmitted intensity, and subsequently averaged using the image analysis software Foxtrot (SWING beamline at SOLEIL Synchrotron). 

The SAXS data were averaged and processed by standard procedures using PRIMUS [20]. The forward scattering *I*(0) and the radii of gyration *R*_*g*_ were evaluated using the Guinier approximation assuming that at very small angles (*s* < 1.3/*R*_*g*_) the intensity is represented as *I*(*s*) = *I*(0)exp⁡(−(*sR*_*g*_)^2^/3). These parameters were also computed from the entire scattering pattern using the indirect transform package GNOM [21], which also provides the maximum dimension of the particle *D*_max⁡_ and the distance distribution function *P*(*r*). Low resolution shape analysis of the solutes was performed using the *ab initio* program DAMMIF [22]. The scattering from the atomic models was calculated using the program CRYSOL [23] which either predicts theoretical scattering patterns or fits the experimental data by adjusting the excluded volume and the contrast of the hydration layer. The program SASREF [24] was employed for molecular rigid body modeling of the PPAR*γ*ΔNTD/RXR*α*ΔNTD/DNA complex, based on the crystal structure of PPAR*γ*/RXR (PDB ID: 3DZY). For both *ab initio* and rigid body analysis, multiple runs were performed to verify the stability of the solution, and the most typical reconstructions were selected using the programs DAMAVER [25] and SUPCOMB [26]. 

## 3. Results and Discussion

### 3.1. Characterization of PPAR*γ*/RXR*α* Complexes

PPAR*γ*/RXR*α* LBDs or full-length proteins were copurified in two steps as described [16, 18]. DNA from *Cyp4A1* regulated gene was added in a 1.2-fold excess to the full-length heterodimer and the complex further purified by analytical gel-filtration. In order to investigate the binding of TIF2 RID on PPAR*γ*/RXR*α*, a 15 kDa fragment of TIF2 (632–772) containing the three LXXLL motifs and interacting with NRs (Figure 1(a)) was added to the heterodimer and further purified by gel-filtration. After gel-filtration, the complexes were analyzed by SDS-PAGE and native electrophoresis confirming the formation and homogeneity of the complexes (see Supplementary Figure 1 available online at doi:10.1155/2012/701412). The stoichiometry of the TIF2 RID/PPAR*γ*/RXR*α* complexes was determined using analytical ultracentrifugation (AUC) and electrospray ionization mass spectrometry (ESI-MS), a method that has proven its efficiency for the study of noncovalent complexes [27, 28]. ESI-MS experiments were performed under nodenaturing conditions on PPAR*γ*/RXR*α* LBDs in the presence of TIF2 RID. The molecular mass obtained (Figure 1(b)) is consistent with a complex composed of one TIF2 RID molecule per heterodimer. The presence of mass peaks corresponding to free PPAR*γ*/RXR*α* LBDs is due to partial dissociation of the complex in the ESI ion source. Equilibrium sedimentation AUC experiments were also carried out to characterize the association state of TIF2 RID with PPAR*γ*ΔNTD/RXR*α*ΔNTD/DNA in solution as well as its molecular mass (Figure 1(c)). The molecular mass of Mw of 114 kDa is in excellent agreement with the expected value of 114.3 kDa. Both measurements indicate that only one TIF2 RID molecule binds to PPAR*γ*/RXR*α* heterodimer. 

### 3.2. Topology of PPAR*γ*/RXR*α* LBDs and TIF2 RID/PPAR*γ*/RXR*α* LBDs Complexes

 The observed stoichiometry of one TIF2 RID per heterodimer contrasts with the crystallographic structures of NRs bound to LXXLL peptides [11–13] which have revealed 2 peptides bound by each monomer of the dimer. This raises the question of the binding mode of TIF2 RID to PPAR*γ*/RXR*α* either asymmetrically to one monomer or to the two subunits involving two NR boxes. To address this question we used SAXS, a structural method to characterize multidomain proteins and complexes. We analyzed by SAXS the recruitment of the TIF2 RID by the PPAR*γ*/RXR*α* LBDs. 

**Figure 2:**
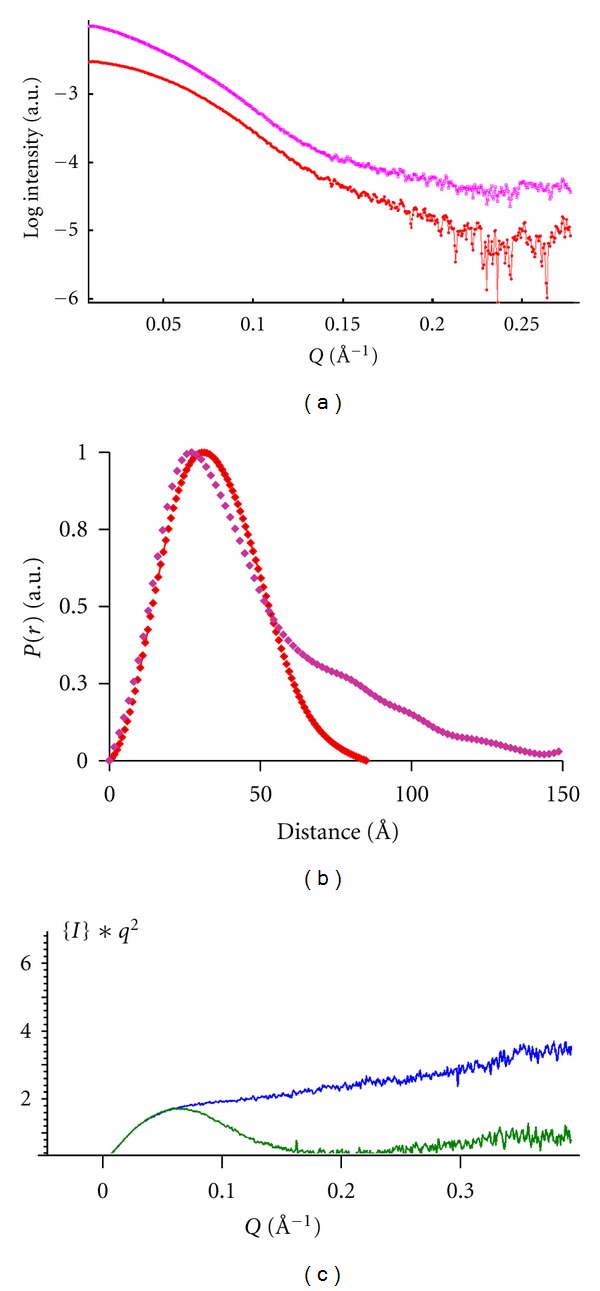
Experimental SAXS data of PPAR*γ*/RXR*α* LBDs complexes. (a) Scattering profiles of PPAR*γ*/RXR*α* LBDs (red) and TIF2 RID/PPAR*γ*/RXR*α* LBDs (pink). (b) Distance distribution functions computed from the X-ray scattering patterns using the program GNOM. Same color code as in (a). (c) Kratky representations for TIF2 RID (blue) and TIF2 RID/PPAR*γ*/RXR*α* LBDs (green).

As a reference, scattering profiles of PPAR*γ*/RXR*α* LBDs alone were collected (Figure 2(a)). Monodisperse concentrated solutions of PPAR*γ*/RXR*α* LBDs were measured, and the structural parameters including the radius of gyration (*R*_*g*_) and the maximum particle dimension (*D*_max⁡_) were computed from the experimental scattering patterns from the heterodimer (Table 1). A symmetrical pair-distribution function is observed (Figure 2(b)) indicating a globular complex. The experimental SAXS data is well fitted by the scattering profile calculated from the crystal structure (PDB ID: 3H0A) using CRYSOL [23] and is in agreement with SAXS parameters measured for heterodimer LBDs [17, 18].

We next analyzed the TIF2 RID/PPAR*γ*/RXR*α* LBDs complex. The values of *R*_*g*_ and *D*_max⁡_ calculated from the distance pair-distribution function *P*(*r*) for TIF2 RID are larger (Table 1) than typical values expected for globular proteins suggesting that TIF2 RID is extended. To have an indication of TIF2 RID folding, we used the Kratky representations, which emphasize deviation from the high-*q* behavior of the scattering intensity *I*(*q*). The Kratky plot of TIF2 RID alone (Figure 2(c)) shows a continuously increasing curve that indicates an unfolded protein. For the TIF2 RID/PPAR*γ*/RXR*α* LBDs complex, it corresponds to a partially folded protein with a bell-shaped curve in agreement with a folded macromolecule and thus suggesting an induced folding of the molecule upon complex formation. Binding of TIF2 RID to the heterodimer led to an increase of the *R*_*g*_ (by 8 Å) and of the *D*_max⁡_ (by 65 Å) (Table 1). The large difference in *R*_*g*_ and *D*_max⁡_ compared to those of PPAR*γ*/RXR*α* LBDs suggests that the TIF2 RID is extended and partially disordered. This is also observed in the asymmetric profile of the pair-distribution function with a tail at high *r* values (Figure 2(b)) which corresponds to elongated molecules in contrast to the symmetric pair-distribution function of the globular PPAR/RXR LBDs. 

**Figure 3:**
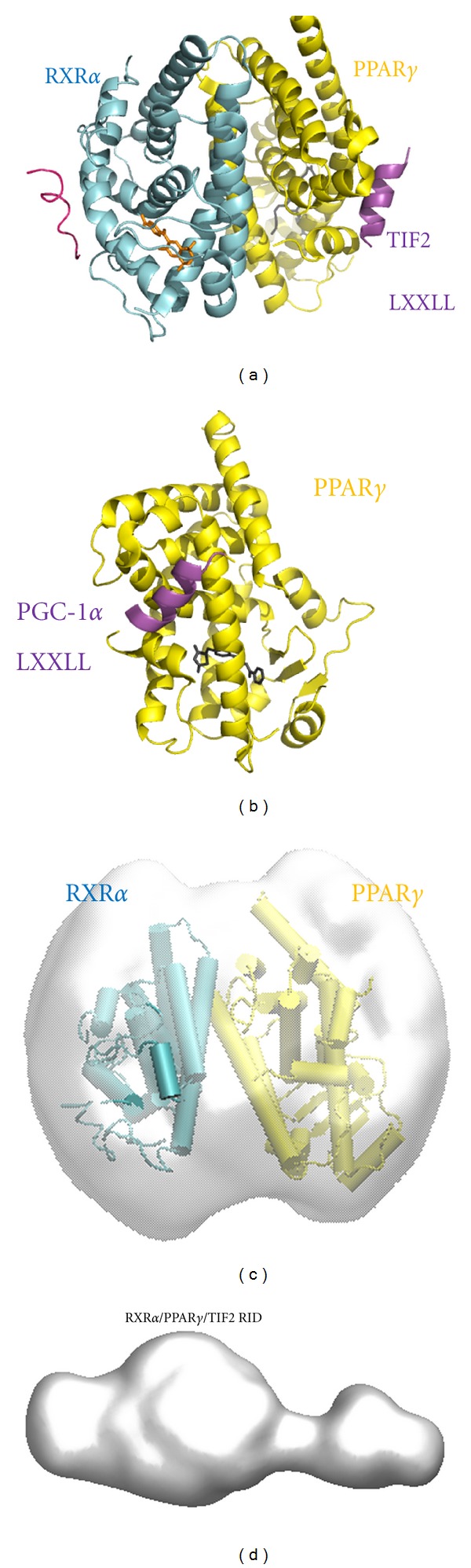
Molecular envelope of PPAR*γ*/RXR*α* LBDs complexes and comparison with the crystal structure of PPAR*γ*/RXR*α* LBDs. (a) Crystal structure of PPAR*γ*/RXR*α* LBDs in complex with TIF2 coactivator peptide (PDB ID: 1H0A) shown in schematic cartoon representations with PPAR*γ* in yellow, RXR*α* in cyan, and the coactivator peptides in pink. (b) Crystal structure of PPAR*γ* LBD bound to PGC-1*α* NR2 motif (PDB ID: 3CS8). (c) Molecular envelopes of the complexes PPAR*γ*/RXR*α* LBDs (grey surface) together with the crystal structure of the complex. (d) Molecular envelope of the complexes TIF2 RID/PPAR*γ*/RXR*α* LBDs (grey surface).

The molecular envelopes as bead models, derived from the SAXS data, were computed for PPAR*γ*/RXR*α* LBDs and TIF2 RID/PPAR*γ*/RXR*α* LBDs (Figure 3). It corresponds to a symmetrical globular shape for the LBD dimer (Figure 3(c)). In contrast, for the TIF2 RID complex, a marked asymmetry as compared to the LBD dimer is observed (Figure 3(d)). In analogy with the TIF2 RID and SRC-1 RID complexes with RAR/RXR and RAR homodimer [16, 29], we can speculate that the globular region of the molecular envelope corresponds to the LBD dimer and the asymmetric extended tail to TIF2 RID. The large difference in the structural parameters of the TIF2 RID complex compared to the LBD dimer and the molecular envelope of the complex suggests that TIF2 RID is flexibly attached asymmetrically to the PPAR*γ*/RXR*α* through only one LXXLL motif. Importantly, the data preclude a model in which two LXXLL motifs bind to each subunit of the PPAR*γ*/RXR heterodimer. A preliminary SAXS study of PPAR*γ*/RXR*α* bound to PGC-1*α* RID provides similar results with similar *R*_*g*_ and *D*_max⁡_ values suggesting that in both cases the architecture of the two different complexes is similar with the CoA RID asymmetrically bound to PPAR subunit through only one LXXLL motif. Interestingly, it has been shown that NR2 motif of PGC-1*α* is sufficient to have a full transcriptional response by PPAR*γ*/RXR*α* [14]. While for TIF2, it has been shown that the third LXXLL motif binds preferentially to PPAR*γ* [14] and a combination of LXXLL motifs is required for a full transcriptional response [30]. However the SAXS data are in agreement with the crystal structure of PPAR*γ*/RXR*α* LBDs (PDB ID: 3H0A) that reveals one coactivator peptide tightly bound to PPAR*γ* and one coactivator peptide loosely bound to RXR*α* as indicated by the poor electron density and high B factor for the peptide bound to RXR (averaged B factor for the peptide bound to RXR is 116.2 compared to the averaged B factor for the CoA peptide bound to PPAR*γ*, 31.6). These observations demonstrate that TIF2 RID binds asymmetrically to PPAR*γ* subunit within the heterodimer through one LXXLL.

### 3.3. Solution Structure of PPAR*γ*2/RXR*α*/Cyp4A1 DR1 Complexes

**Figure 4:**
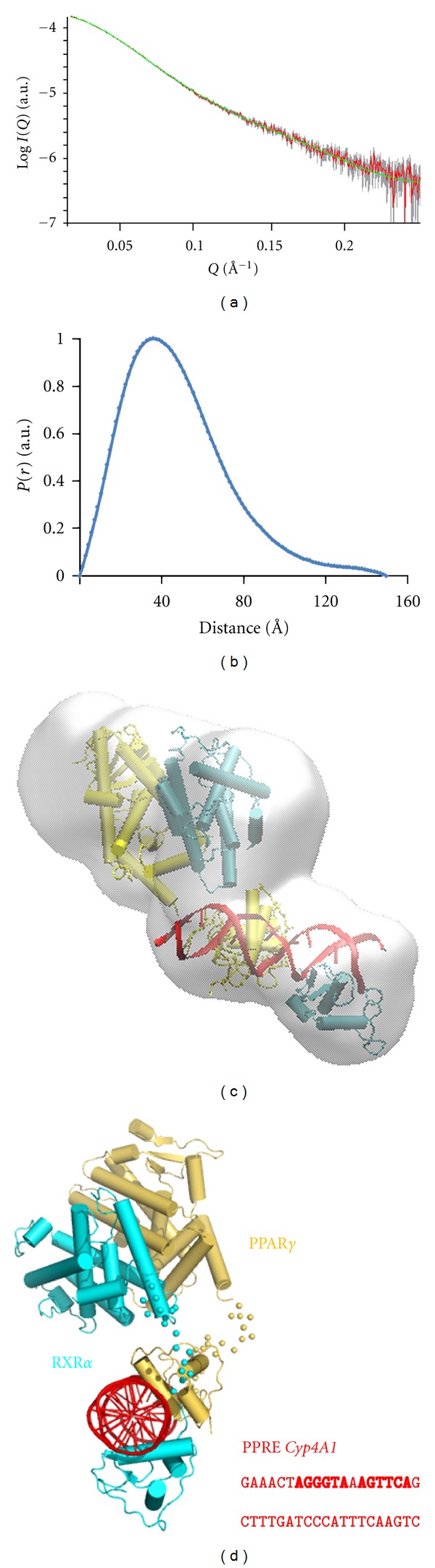
Solution structure of PPAR*γ*2/RXR*α* bound to *Cyp4A1* PPRE. (a) Scattering profiles of PPAR*γ*2ΔNTD/RXR*α*ΔNTD/PPRE. Experimental data are shown as red dots. Green fit is computed from the solution structure of the complex. (b) Distance distribution function computed from the X-ray scattering pattern using the program GNOM. (c) Most typical molecular envelope of PPAR*γ*2ΔNTD/RXR*α*ΔNTD/PPRE generated by DAMMIF (beads model shown as a grey surface) together with the refined model by rigid body refinement using the program SASREF (fit to the experimental data with *χR* = 0.98). (d) Pseudoatomic solution structure of PPAR*γ*2ΔNTD/RXR*α*ΔNTD/*Cyp4A1* PPRE shown in schematic cartoon representation together with the sequence of the DNA.

PPAR*γ*/RXR*α* without DNA has been shown to be elongated with no interdomain interactions [17]. In complex with idealized DNA, the first atomic resolution structure of integral PPAR*γ*/RXR*α* obtained by X-ray crystallography [15] confirmed the structural information concerning isolated domains [11, 31, 32] and also revealed an interdomain contact between the LBD of PPAR*γ* and the DBD of RXR [13], although the functional correlation is limited to a single point mutation. The N-terminal domain, unfolded, was not visible in the electron density map. We previously measured the SAXS profile of PPAR*γ*2/RXR*α* bound to an idealized DR1 [16] and have shown that the conformation in solution is different to that observed in the crystal structure with the heterodimer exhibiting an extended asymmetric shape without additional interdomain contacts between the DBDs and LBDs beyond the connection through the hinge regions. We have now characterized the solution structure of PPAR*γ*2/RXR*α* full length or truncated of their NTDs and bound to a natural PPRE from *CYP4A1*. The structural parameters calculated from the experimental scattering patterns (Figures 4(a) and 4(b)) given in Table 1 reveal an extended shape of the complex. With the full length PPAR*γ* complex, the structural parameters are even larger (*R*_*g*_ = 44 Å and *D*_max⁡_ = 160 Å), suggesting a distinct dissociated NTD. Low resolution models were reconstructed *ab initio* from the corresponding experimental scattering patterns. The most typical *ab initio* model of PPAR*γ*2ΔNTD/RXR*α*ΔNTD/*CYP4A1* PPRE presented in Figure 4(c) clearly displays separate DBD and LDB domains similar to the architecture observed for the complex with the idealized DR1 and those observed for other NR heterodimers. For PPAR*γ*2ΔNTD/RXR*α*ΔNTD/PPRE, its atomic structure was refined against SAXS experimental data (Figure 4(a)). The position of the domains was adjusted by rigid body modeling using the available high resolution crystal structure of the complex (PDB ID: 3DZY). The model obtained by rigid body refinement agrees well with the *ab initio* models as seen from the superposition in Figure 4(c). The refined structure reveals an asymmetric shape with the LBD dimer positioned at the 5′ end of the DNA (Figure 4(d)), an asymmetry already observed for other heterodimers studied by SAXS [16]. 

The scattering patterns of the full-length PPAR*γ*2/RXR*α*/PPRE in complex with TIF2 RID were also monitored. The increase of the *R*_*g*_ and of the *D*_max⁡_ (Table 1) upon TIF2 RID interaction to the full-length heterodimer is similar to that observed for the complex with the LBD dimer, suggesting a similar mode of binding as the LBD dimer with no additional interactions of the TIF2 RID with the full-length complex. The solution studies of DNA complexes with integral receptors provided also evidence for a stoichiometry of 1 coactivator per receptor dimer. Each heterodimer binds only one coactivator protein via PPAR, this preferential binding being controlled by affinity, rather than steric exclusion. However, other domains of TIF2 may be implicated in additional interactions to the receptor as TIF2 has been shown to bridge the N-terminal and C-terminal receptor domains of estrogen receptor [33].

## 4. Conclusion

The solution structures of the functional heterodimer PPAR*γ*2/RXR*α* bound to natural DR1 from regulated gene and TIF2 RID reveal the asymmetry induced by the nonsymmetric DNA target of the NR dimer with the position of the LBDs at the 5′ end of the target DNA and the asymmetric mode of recruitment of TIF2. The extended conformation in solution of integral PPAR*γ*/RXR*α* bound to DNA is recognized and maintained during coactivator binding. The TIF2 RID, which is intrinsically disordered, partially folds on binding to PPAR*γ* but retains an extended conformation in the complex. The functional consequence imposed by the DNA is the asymmetric binding of the cofactor with the complex that orients the coactivator on one side of the DNA, allowing interactions with other regulatory proteins and thus leading to specific and controlled NR-mediated gene transcription. As other domains outside the TIF2 RID may interact with the heterodimer, further studies should be carried out to characterize the topology of such large complexes. A better understanding at the molecular level of the regulation of PPAR*γ*/RXR*α* by their coactivators would benefit new therapeutic approaches for the treatment of metabolic diseases.

## Supplementary Material

Supplementary Figure 1 with the coomassie-stained SDS-PAGE and Native-PAGE of the PPARg2/RXRa complexes after the final purification step. (a) 12% SDS gel for PPARg2/RXRa complex, (b) 12% SDS gel (left) and 8-25% Native gel for PPARg2/RXRaDeltaNTD/DNA, (c) 12% SDS gel for PPARg2DeltaNTD/RXRaDeltaNTD/DNA/TIF2 RID and (d) 12% SDS gel for PPARg LBD/TIF2 RID complex.

## Figures and Tables

**Table 1:** Structural parameters from SAXS data.

Complexes	*R*_*g*_ (Å)	*D*_max⁡_ (Å)
PPAR*γ*/RXR*α* LBD	27.2 ± 0.1	85 ± 5
TIF2 RID/PPAR*γ*/RXR*α* LBD	35.5 ± 0.1	150 ± 10
TIF2 RID	33 ± 1	130 ± 10
PPAR*γ*2ΔNTD/RXR*α*ΔNTD/PPRE (CYP4A1)	37.1 ± 0.4	140 ± 10
PPAR*γ*2/RXR*α*ΔNTD/PPRE	44 ± 0.5	160 ± 10
PPAR*γ*2/RXR*α*/PPRE	52 ± 0.5 [16]	180 ± 10 [16]
TIF2 RID/PPAR*γ*2/RXR*α*/PPRE	59 ± 0.5	200 ± 20
