# Supplementary material for: Solution Structures of PPARγ2/RXRα Complexes
Source: PPAR Res. 2012 Dec 18;2012:701412. doi: 10.1155/2012/701412 (PMC3539437; doi:10.1155/2012/701412)

**Supplementary Figure 1: Coomassie-stained SDS-PAGE and Native-PAGE of the PPARγ2/RXRα complexes after the final purification step.**


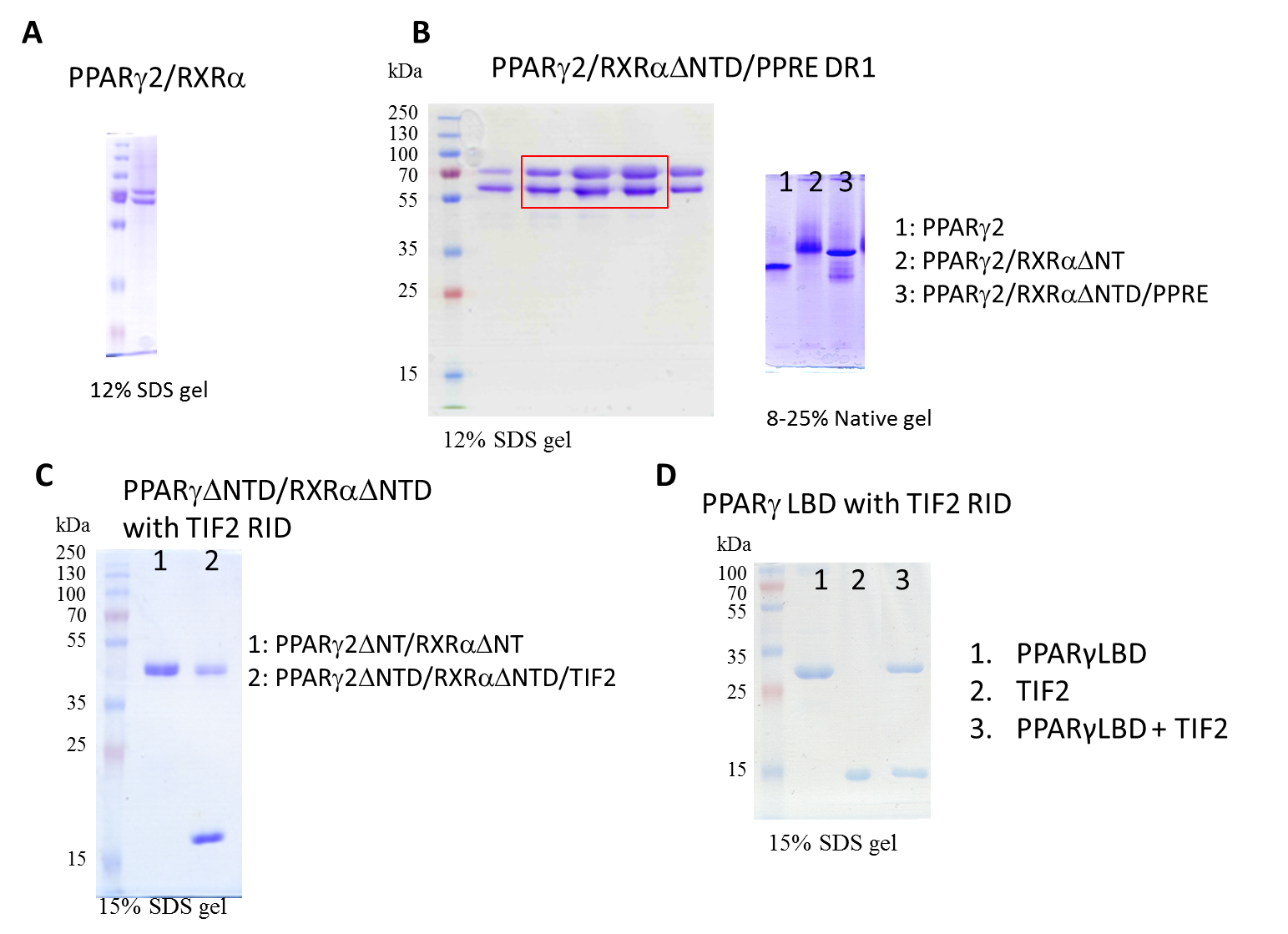

Supplement: Supplementary file 1 — Supplementary Figure 1 with the coomassie-stained SDS-PAGE and Native-PAGE of the PPARg2/RXRa complexes after the final purification step. (a) 12% SDS gel for PPARg2/RXRa complex, (b) 12% SDS gel (left) and 8-25% Native gel for PPARg2/RXRaDeltaNTD/DNA, (c) 12% SDS gel for PPARg2DeltaNTD/RXRaDeltaNTD/DNA/TIF2 RID and (d) 12% SDS gel for PPARg LBD/TIF2 RID complex. [file 701412.f1.docx]
